# What the Dying are Saying to the Living: Mixed Conspecific Alarm Cues Reverse Competitive Hierarchies by Reshaping Genotype‐Specific Life Histories

**DOI:** 10.1002/ece3.74356

**Published:** 2026-09-13

**Authors:** Xiaofei Tian, Qi Zhang, Cheng Li, Wenping Feng, Xiumei Zhang

**Affiliations:** ^1^ Fishery College Zhejiang Ocean University Zhoushan China

**Keywords:** alarm cues, chemical interference, competitive hierarchy, *daphnia*, life‐history strategy, stress response

## Abstract

A central principle of chemical ecology theory posits that reliable information about predation risk, conveyed through conspecific alarm cues, enables adaptive phenotypic plasticity. While responses are known to be finely tuned to predator identity and diet, the consequences of conflicting information, specifically from mixed alarm cues of genetically distinct conspecifics, remain unexplored. We test the novel hypothesis that such mixed cues constitute “chemical interference,” which disrupts adaptive decision‐making by overriding canonical plastic responses. Using two obligate parthenogenetic *Daphnia* genotypes as a model system, we compared life‐history responses to exposure to genotype‐specific (single) versus mixed‐genotype alarm cues and linked these responses to outcomes from long‐term intraspecific competition experiments. While single cues triggered the canonical adaptive “life‐history switch,” characterized by accelerated growth and reproduction, mixed cues did not amplify this response. Instead, they induced a state of chronic stress, leading to severe, genotype‐specific suppression of growth and reproduction and a phenotypic restructuring toward a conservative, slow‐growth strategy. This phenotypic reprogramming was associated with altered competitive outcomes. The genotype (A5) whose mixed‐cue phenotype was less constrained in its growth trajectory showed strong competitive performance, contributing to a shift in the competitive hierarchy compared to single‐culture controls. In contrast, the other genotype (A1) suffered significant competitive suppression. Our findings demonstrate that the informational content of alarm cues (pure vs. mixed) acts as a critical environmental filter, inducing divergent, genotype‐specific life‐history strategies. This leads to a context‐dependent reversal of competitive dominance, where each genotype's fitness is maximized under a specific informational scenario. This mechanism provides a novel, non‐genetic explanation for how variation in chemical information flow can contribute to the maintenance of genetic and phenotypic diversity within populations facing uncertain risks.

## Graphical abstract

This study demonstrates that the complexity of intraspecific alarm cues—specifically, whether they are from a single genetic source or a mixture—acts as a critical environmental filter. While genotype‐specific cues trigger a canonical adaptive “life‐history switch,” mixed cues induce a state of chronic stress, leading to genotype‐specific reprogramming of growth and reproduction. This phenotypic restructuring, in turn, causes a context‐dependent reversal of competitive dominance between genotypes, revealing a novel pathway by which chemical information flow can maintain genetic diversity.

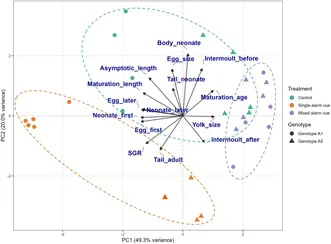

## 1 Introduction

Intraspecific communication via chemical cues is a fundamental mechanism mediating ecological interactions, spanning from predator avoidance to resource competition (Bailey et al. 2006; Barki et al. 2010; Kamio et al. 2022). Among these cues, alarm signals that are released upon injury or predation play a critical role in inducing defensive phenotypic plasticity in conspecifics (Schoeppner and Relyea 2005; Brönmark and Hansson 2012). In aquatic ecosystems, such chemical communication is particularly prevalent. Cladocerans like *Daphnia* exhibit a range of inducible life‐history and morphological responses to such chemical information (Tollrian and Harvell 1999). Notably, these responses are not triggered by predator identity alone but are often finely tuned to the specific diet of the predator, with cues from predators fed phylogenetically related prey eliciting stronger defenses (Dodson 1988; Stabell et al. 2003; Gu et al. 2023). This dependency on prey identity underscores that the information content of chemical cues, whether they signal a clear, specific threat or a generalized risk, is crucial for adaptive decision‐making.

The canonical adaptive response to a reliable predation threat, often termed the “life‐history switch,” typically involves accelerated development and increased early reproduction, theoretically enhancing fitness under predation risk (Boersma et al. 1998; Lass and Spaak 2003; Stoks et al. 2016). This phenomenon highlights the adaptive significance of receiving reliable information. However, natural environments are informationally complex. Prey are often exposed to a blend of chemical cues originating from multiple sources, including different conspecifics or heterospecifics within a prey community (Relyea 2003). While studies have explored how cue complexity across predator identities shapes prey responses (e.g., Schoeppner and Relyea 2005), a critical gap remains at the intraspecific level: in genetically diverse populations, a common state in both sexual and asexual species (Hebert 1982, 1987), how do prey interpret and respond to a blend of alarm cues emanating from genetically distinct conspecifics?

In nature, competitors are embedded in a complex web of biotic interactions, and competitive outcomes themselves can be a source of mortality (Sommer 2002; Holmes et al. 2016). As individuals compete and die, they release conspecific alarm cues into the shared environment. Given the spatial and temporal patchiness of ecological events, the cue landscape experienced by an individual likely varies along a continuum from being dominated by signals from a single, locally abundant genotype to a complex mixture from many. The key question, therefore, is not whether mixed cues exist, but how the degree of cue complexity (i.e., informational clarity versus noise) shapes phenotypic decisions. Does a mixture act as a reinforced, reliable signal of intense, generalized risk, prompting a unified defensive response? Or does it create informational conflict or “chemical noise” that disrupts the typical adaptive plasticity, potentially inducing a state of chronic stress? Conflicting information can lead to constrained or even maladaptive phenotypes (Ferrari et al. 2010), and the life‐history strategies induced under such conflict could have direct consequences for competitive ability.

The outcome of this informational conflict may hinge on how different genotypes manage the fundamental life‐history trade‐off between reproduction and somatic maintenance under stress. Theoretical models predict that under chronic or uncertain stress, optimal strategies may shift from maximizing immediate reproductive output to securing somatic persistence and efficient resource utilization (Watts and Tenhumberg 2021; Thunell et al. 2022). Consequently, a genotype's competitive fate under informational conflict may be less about the magnitude of a single defensive trait and more about the integrated efficiency of its stress‐induced resource allocation strategy. For example, a genotype that accelerates reproduction under a clear alarm cue might gain a short‐term numerical advantage, but the same strategy could be metabolically costly under the chronic stress simulated by a mixed cue, potentially rendering it competitively inferior to a genotype that maintains somatic growth and turnover.

Here, we address this knowledge gap by integrating chemical information ecology with competitive dynamics. We use two obligate parthenogenetic genotypes of the water flea *Daphnia* cf. *pulex* as a model system. This clonal system allows us to experimentally deconstruct the natural cue blend, isolating the effects of cue genetic identity and complexity. We first ask: how does exposure to a blend of alarm cues from different genotypes (a mixed cue) compare to exposure to a genotype‐specific alarm cue (a single cue) in shaping life‐history traits? We hypothesize that the mixed cue will not simply average the responses but will create a signal of high uncertainty, overriding the canonical adaptive life‐history switch and inducing a state of chronic stress. We predict fundamentally different phenotypic outcomes: accelerated life history under the single cue versus suppressed growth and reproduction under the mixed cue. Furthermore, we posit that these cue‐induced phenotypic shifts could directly alter the outcomes of intraspecific competition. To test this functional link, we connect the detailed life‐history phenotypes induced under different cue environments to the results of a separate, long‐term competition experiment between the same genotypes. We predict that the competitive hierarchy will be contingent upon the informational context, favoring the genotype whose induced phenotype is more robust in that specific context. Therefore, this study tests the novel hypothesis that mixed intraspecific alarm cues act as a form of ‘chemical interference,’ disrupting adaptive decision‐making, triggering genotype‐specific stress responses that reprogram resource allocation, and thereby altering competitive dynamics through phenotypic plasticity.

## 2 Methods and Materials

Two obligate parthenogenetic genotypes (A1, A5) of the *Daphnia* cf. *Pulex* JPN1 lineage were selected for this study. This experimental system provides a genetically invariant background within each clonal line, allowing us to isolate and investigate genotype‐specific, inherent capacities for phenotypic plasticity in response to chemical information, rather than measuring adaptations that could emerge in outbred sexual populations. Genotype A1 was originally collected from Lake Hataya Ohnuma (Yamagata Prefecture, Japan: 38.245° N, 140.204° E), and A5 from Furuichi Oike Pond (Tottori Prefecture, Japan: 35.391° N, 133.339° E) (So et al. 2015; Tian et al. 2019, 2024). Clonal stock cultures of each genotype have been maintained under standardized laboratory conditions (20°C, 14:10 h light: dark cycle) in COMBO freshwater medium (Kilham et al. 1998) for several years. The use of such long‐term, stable clones ensures phenotypic consistency and enables the precise deconstruction of how cue complexity (single‐ vs. mixed‐genotype alarm signals) shapes life‐history strategies in a controlled setting. The green alga *Scenedesmus obliquus* , cultured in a flow‐through system with COMBO medium, served as the food source. Algal carbon content was determined to be 2.09 × 10^−8^ mg C per cell. All experimental animals were fed *S. obliquus* at a concentration of 2.0 mg C L^−1^ daily. The medium was completely renewed every other day, maintaining a density of < 1 individual per 20 mL. Neonates born within 24 h from the third brood of these stock cultures were used to initiate all experiments.

### 2.1 Alarm Cue Preparation and Life‐History Assay

Two types of chemical cues were prepared. For the single alarm treatment, eight 5‐day‐old individuals from a single genotype (A1 or A5) were homogenized in 1.0 mL of COMBO medium using a micropestle. The homogenate was centrifuged at 1000 × g for 5 min. The resulting supernatant, containing genotype‐specific alarm cues, was defined as the stock alarm cue solution. For the mixed alarm treatment, equal volumes of the A1 and A5 stock cue solutions were combined. All cue preparations were used within 1 h of preparation and were not subjected to freeze–thaw cycles.

For the life‐history experiment, neonates (0–24 h old) of each genotype were individually placed into 50 mL stoppered bottles containing COMBO medium with *S. obliquus* at 2.0 mg C L^−1^. The experiment was conducted at 20°C. For each genotype, 15 individuals were randomly and equally assigned to one of three treatments: control (COMBO medium only), single alarm (exposed to the cue from their own genotype), or mixed alarm (exposed to the mixed‐genotype cue). The culture medium was completely renewed every day. Immediately following each renewal, the alarm cue exposure was maintained by removing 1.0 mL of the fresh medium and replacing it with 1.0 mL of the corresponding fresh cue supernatant. Animals were fed immediately after this procedure. The experiment continued until the production of the third brood.

During each medium change, animals were photographed using a stereomicroscope (Olympus CX43) equipped with a digital camera. Body length (from the base of the tail spine to the top of the head), tail spine length, and the size and number of eggs in the brood pouch were measured from images using ImageJ software. Maturation was defined as the day eggs first appeared in the brood pouch. The carapace length and width of at least three eggs per clutch were measured to estimate yolk volume (Tian et al. 2019). The number of molted exuviae and the body length of newly released neonates were also recorded at each census.

The Specific Growth Rate (*SGR*) for the first 5 days was calculated as follows:

$$\mathrm{SGR}\;\left({\mathrm{day}}^{-1}\right)=\left(\ln\left[{\mathrm{BL}}_{5}\right]–\ln\left[{\mathrm{BL}}_{0}\right]\right)/5,$$

where *BL*_*0*_ and *BL*_*5*_ are body lengths at Days 0 and 5, respectively. The asymptotic body length (*L∞*) and growth coefficient (*k*) for each individual were estimated by fitting its growth data to the von Bertalanffy growth function:

$$L_{t}=L\infty \times \left\{1–\exp\;\left[–k\;\left(t–t_{0}\right)\right]\right\},$$

where *L*_*t*_ is body length at age *t* (days), and *t*_*0*_ is the hypothetical age at zero length.

### 2.2 Competition Dynamics Data

To investigate the consequences of life‐history shifts for intraspecific competition, we utilized population time‐series data from a previously published 60‐day competition experiment (Tian et al. 2022). Briefly, that experiment was conducted in 1 L bottles containing COMBO medium with *S. obliquus* . It included single‐genotype controls (20 individuals of one genotype) and two‐genotype competition treatments (10 individuals each of A1 and A5). Populations were maintained at 20°C with gentle daily mixing and periodic medium renewal. Food was replenished every third day, maintaining an approximate average supply of 0.33 mg C L^−1^ day^−1^. Population abundance and the relative proportion of each genotype (determined via genetic analysis) were censused on Days 12, 36, and 60. For the present study, we extracted the time‐series data (population size and relative proportion) for the A1 vs. A5 two‐genotype competition runs and their corresponding single‐genotype controls for Days 0, 12, 36, and 60. This allowed us to test for associations between the life‐history phenotypes induced in our cue‐exposure experiment and the competitive outcomes observed at the population level. In this design, the single‐genotype controls correspond to a single‐alarm‐cue environment, where alarm cues released through natural mortality originate from a single genotype, whereas the two‐genotype competition treatment corresponds to a mixed‐alarm‐cue environment, where alarm cues originate from both genotypes.

### 2.3 Statistical Analysis

For each of the 15 measured life‐history traits, a two‐way ANOVA was performed to test the effects of Genotype (A1, A5), Treatment (Control, single alarm, mixed alarm), and their interaction (Model: Trait ~ Genotype × Treatment). Data were checked for normality (Shapiro–Wilk test) and homogeneity of variances (Levene's test). When assumptions were not met, data were transformed (e.g., log‐transformation for maturation age). After transformation, all traits satisfied parametric test assumptions. Pairwise comparisons within each trait were conducted using Tukey's HSD test. To control for multiple testing across the 15 traits, the Benjamini‐Hochberg false discovery rate (FDR) correction was applied to the ANOVA main effects and interaction terms. All statistical tests were two‐tailed, and significance was set at *α* = 0.05. Afterwards, a principal component analysis (PCA) was conducted on the correlation matrix of the 15 life‐history traits to reduce dimensionality and visualize the primary patterns of multivariate phenotypic variation across treatments and genotypes.

For population trajectories under competitive conditions, we employed a multi‐tiered analytical approach. First, for each genotype, the competition effect size at each time point (Day 12, 36, 60) was calculated as follows:

$$\text{Effect}\;\left(\%\right)=\left[\left(\text{Population size in competition}–\text{Mean population size in single culture}\right)/\text{Mean population size in single culture}\right]\times 100,$$

where the mean population size in single culture was calculated across all single‐culture replicates of the same genotype at the same time point. Since the competition and single‐culture replicates were not paired, each competition replicate was compared to this common baseline mean. To determine whether competition had a significant effect on each genotype at a given time, we performed independent samples *t*‐tests. For each genotype (A1 and A5) separately, we compared the population size between the single culture and competition treatments at Days 12, 36, and 60. Second, to test for a reversal in the competitive hierarchy over time, we fitted a linear mixed‐effects model to the time‐series data. The log10‐transformed population size (abundance) was used as the response variable. The fixed‐effect predictors were: Genotype (A1 or A5), Condition (‘single culture’ as the control, and ‘competition’ for the mixed‐genotype treatment), and Day (12, 36, 60), along with all possible two‐way and the three‐way interaction terms. The replicate bottle identity was included as a random intercept (1|Replicate) to account for repeated measures. The full model was specified as:

$$\begin{array}{c} \log_{10}\left(\text{Population size}+1\right)\sim \text{Genotype}+\text{Condition}+\mathrm{Day}+\text{Genotype}\\ \times \text{Condition}+\text{Genotype}\times \mathrm{Day}+\text{Condition}\times \mathrm{Day}\\ +\text{Genotype}\times \text{Condition}\times \mathrm{Day}+\left(1|\text{Replicate}\right). \end{array}$$

A significant three‐way interaction (Genotype × Condition × Day) would indicate genotype‐specific responses to competition over time.

To identify which life‐history traits might mediate the observed reversal in competitive dominance under mixed alarm cues, we conducted a targeted analysis. First, we performed a two‐way ANOVA (Model: Trait ~ Genotype × Treatment) using only the data from the Control and Alarm‐mixed treatments. Traits exhibiting a statistically significant Genotype × Treatment interaction (*p* < 0.05) in this model were selected as candidates, indicating that the genotypic difference for that trait was contingent upon the cue environment. For each candidate trait, we then calculated the genotype mean difference under mixed alarm cues, defined as:

$$\text{Trait Difference}=\text{Mean}\;\left(\text{A1 under mixed cues}\right)–\text{Mean}\;\left(\text{A5 under mixed cues}\right).$$

The statistical significance of this within‐treatment difference was assessed by testing the simple main effect of Genotype within the Alarm‐mixed treatment level, derived from the two‐way ANOVA model described above. *p*‐values from these nine tests were adjusted using the Benjamini–Hochberg false discovery rate (FDR) correction to account for multiple comparisons.

To link this phenotypic divergence to the population‐level outcome, we calculated the difference in competitive performance on Day 60 as follows:

$$\begin{array}{cc} \text{Competition Difference}= & \text{Net Competition Effect}\;\left(\text{A1},\mathrm{Day}\;60\right)\\ & –\text{Net Competition Effect}\;\left(\text{A5},\mathrm{Day}\;60\right), \end{array}$$

where Net Competition Effect is the competition effect size for a given genotype, as defined above. A negative value for Competition Difference indicates that genotype A5 was the superior competitor. We then examined whether the sign (direction) of the Trait Difference for each candidate trait aligned with the sign of the Competition Difference. However, because the candidate traits are measured on fundamentally different scales (e.g., maturation age in days vs. yolk size in millimeters), the raw Trait Difference alone is not directly comparable across traits. To allow meaningful cross‐trait comparisons, we standardized the Trait Difference for each trait using Cohen's *d*, calculated as the difference between the A1 and A5 means divided by the pooled standard deviation (SD):

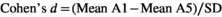




This yields a dimensionless effect size that quantifies the magnitude of phenotypic divergence in standard deviation units. We then integrated both the standardized phenotypic divergence and the competitive outcome into a composite Relevance Score, following the conceptual approach of Kraft et al. (2015):

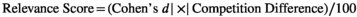




This score provides a relative measure of the strength of association, with higher scores indicating a trait whose divergence under mixed cues is concurrently associated with a larger shift in competitive outcome.

All statistical analyses were performed in R version 4.4.1 (R Core Team 2024). Analyses were conducted using the following R packages: tidyverse (Wickham et al. 2019) for data manipulation, ggplot2 (Wickham 2016) for visualization, lme4 (Bates et al. 2015) and lmerTest (Kuznetsova et al. 2017) for mixed‐effects modeling, car (Fox and Weisberg 2019) for ANOVA, and emmeans (Lenth et al. 2023) for post hoc comparisons.

## 3 Results

### 3.1 Effects of Alarm Cues on Life‐History Traits

Two‐way ANOVA revealed differential effects of genotype, alarm cue treatment, and their interaction on life‐history traits (Table 1). Genotype significantly affected all 15 traits. Alarm cue treatment (Group) had significant effects on 10 out of 15 traits (except for both intermoult rates, tail spine length of neonate, and sizes of egg and neonate). The genotype × treatment interaction was significant for 11 out of 15 traits (except for sizes of egg and neonate, tail spine length of both neonate and adult), indicating genotype‐specific responses of these traits to alarm cues.

**TABLE 1:** The results of two‐way ANOVA with the effects of genotype, alarm cue treatment, and their interaction on phenotypic traits in *Daphnia pulex* . Significant *p*‐values are shown in bold.

Trait	Genotype (G)		Cue treatment (Ct)		G × Ct	
	*F*	*p*	*F*	*p*	*F*	*p*
Maturation age (days)	102.96	**< 0.001**	5.35	**0.037**	15.25	**< 0.001**
Maturation body length (mm)	5054.38	**< 0.001**	37.23	**< 0.001**	48.79	**< 0.001**
Intermoult duration before maturation (days)	620.42	**< 0.001**	4.58	0.052	13.18	**< 0.001**
Intermoult duration after maturation (days)	596.57	**< 0.001**	0.017	0.896	13.07	**< 0.001**
SGR (day^−1^)	362.71	**< 0.001**	15.13	**0.001**	22.31	**< 0.001**
Egg number of the first clutch	200.00	**< 0.001**	12.25	**0.003**	55.50	**< 0.001**
Mean egg number of the second and third clutch	169.28	**< 0.001**	11.56	**0.003**	106.34	**< 0.001**
Neonate number of the first clutch	185.14	**< 0.001**	18.28	**< 0.001**	69.33	**< 0.001**
Mean neonate number of the second and third clutch	163.14	**< 0.001**	17.64	**< 0.001**	103.61	**< 0.001**
Mean egg volume of the first three clutches (mm^3^)	252.34	**< 0.001**	0.89	0.373	0.71	0.510
Yolk‐to‐egg volume ratio in the first three clutches	53.70	**< 0.001**	62.13	**< 0.001**	8.10	**0.003**
Mean neonate size of the first three clutches (mm)	3470.80	**< 0.001**	0.88	0.373	3.52	0.054
Relative tail spine length of the newly born neonate	2243.59	**< 0.001**	3.08	0.103	1.92	0.1834
Relative tail spine length of the first adult instar	1123.52	**< 0.001**	5.64	**0.034**	2.96	0.083
Asymptotic body length (mm)	1355.81	**< 0.001**	15.37	**0.001**	54.92	**< 0.001**

**FIGURE 1:**
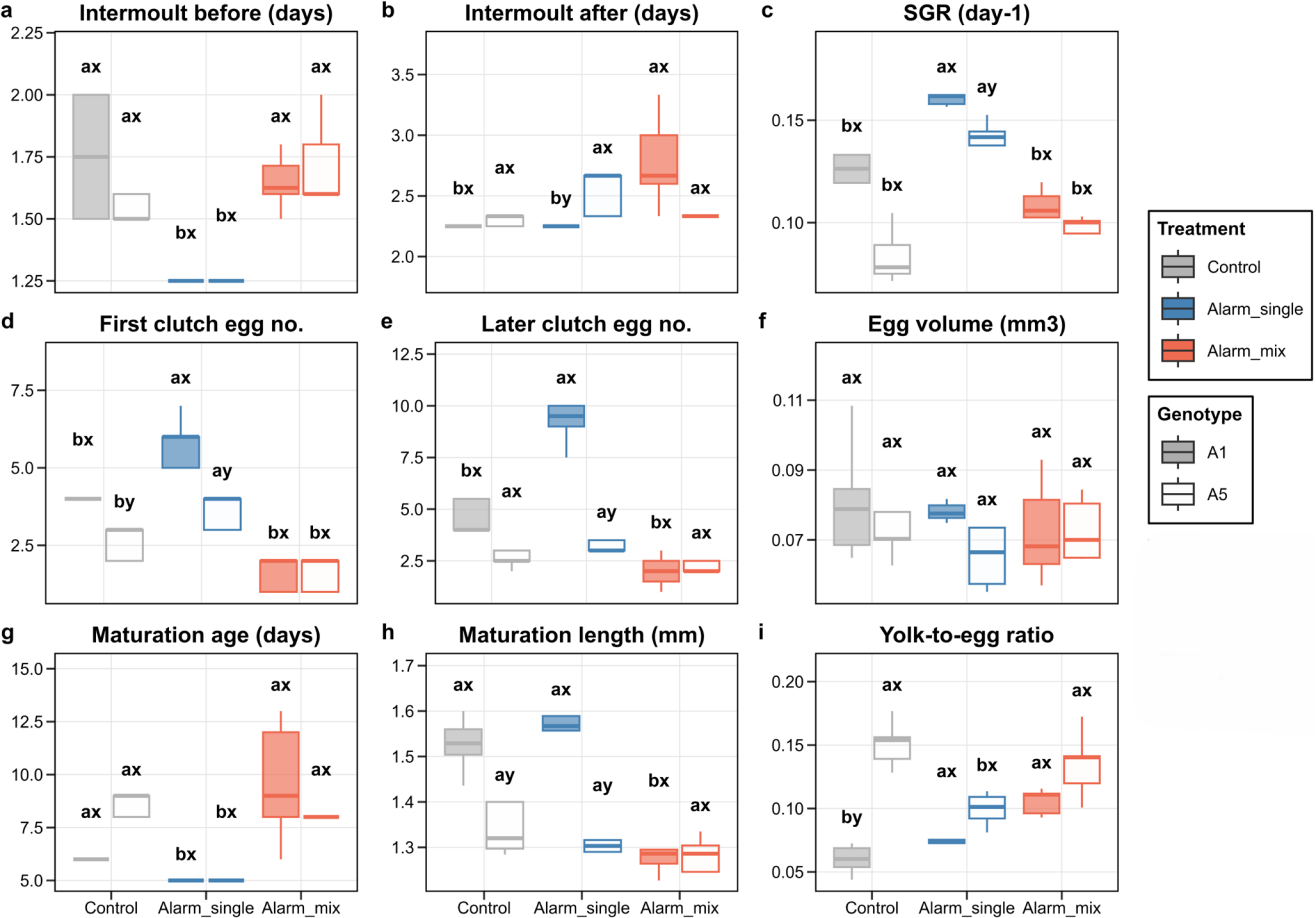
Genotype‐specific plastic responses of *Daphnia* life history traits to different alarm cue environments. The panel displays trait responses (e.g., growth, reproduction, morphology) of two clonal *Daphnia* genotypes (A1 and A5) under control, conspecific alarm cue, and mixed alarm cue conditions. Color denotes treatment (gray: Control; blue: Alarm single; orange: Alarm mix). Filled/open boxes represent genotypes A1/A5, respectively. Within each genotype, different letters (a, b) indicate significant treatment differences; within each treatment, different letters (x, y) indicate significant genotype differences. Same letters indicate no significant difference (FDR‐corrected, *p* > 0.05). (Maturation length: Maturation body length; Intermoult before: Intermoult duration before maturation; Intermoult after: Intermoult duration after maturation; First clutch egg no.: Egg number of the first clutch; Later clutch egg no.: Mean egg number of the second and third clutch; Egg volume: Mean egg volume of the first three clutches; Yolk‐to‐egg ratio: Yolk‐to‐egg volume ratio in the first three clutches). The remaining six traits (Neonate number of the first clutch, Mean neonate number of the second and third clutch, Mean neonate size of the first three clutches, Relative tail spine length of the newly born neonate, Relative tail spine length of the first adult instar, Asymptotic body length) are presented in Figure S1.

Under single alarm cue treatment, both genotypes exhibited adaptive shifts typical of predator‐induced plasticity, albeit with varying magnitudes (Figure 1, S1 and S2). A canonical “life‐history switch” toward accelerated development and reproduction was observed: specific growth rate (SGR) increased significantly (+36.0% in A1, +67.9% in A5), intermoult interval before maturation shortened (−27.7% in A1, −16.7% in A5), and maturation was advanced (age: −19.4% in A1, −34.2% in A5). Concurrently, reproductive investment was heightened, with substantial increases in both early egg production (+45.0% in A1, +38.5% in A5) and later egg production in A1 (+100.0%), whereas the increase in A5 was minimal (+3.5%, non‐significant). This response pattern was significantly different from the control. Direct comparison between the two genotypes under single alarm cues revealed extensive and significant differences: A1 exhibited higher SGR, greater maturation and asymptotic body length, shorter post‐maturation intermoult intervals, and substantially higher reproductive output across all clutch types compared to A5 (all FDR‐corrected *p* < 0.05; Figure 1, Table S2).

Under mixed alarm cue treatment, the phenotypic landscape was fundamentally restructured. The adaptive shift seen under single cues was reversed or suppressed. Most growth and reproductive traits showed significant declines compared to the control, with the magnitude of suppression being more severe in A1 than in A5 (Figure 1, S1 and S2). For instance, SGR was suppressed in A1 (−11.5%), while A5 showed a modest, non‐significant increase (+14.7%). Reproductive traits were strongly and consistently suppressed in A1 (first clutch egg: −60.0%), whereas A5 showed numerically smaller reductions (first clutch egg: −38.5%; later clutch neonate: −21.7%). A1 displayed a pronounced delay in maturation (maturation age: +54.8%), whereas A5 showed no change. The divergent impact on the two genotypes is also evident in the response of asymptotic length, which was severely reduced in A1 (−33.5%) but only moderately reduced in A5 (−17.1%). Despite the pronounced differences in the magnitude of response to mixed cues between A1 and A5 described above, direct pairwise comparisons between the two genotypes under the same treatment condition revealed no statistically significant differences for any of the 15 measured traits after FDR correction.

Critically, a direct comparison of the response ratios revealed profound, strategy‐level genotype‐by‐treatment interactions (Table 1). The adaptive increase in later egg reproduction observed under single cues (A1: +100.0%; A5: +3.5%) was not merely attenuated but reversed under mixed cues, with both genotypes showing suppression that was markedly more severe in A1 (−56.5%) than in A5 (−24.1%). This pattern of differential sensitivity was mirrored in developmental timing, where the acceleration of maturation age under single cues (A1: −19.4%; A5: −34.2%) flipped to a dramatic delay in A1 (+54.8%) under mixed cues, while A5 shifted to a state of no change. The divergent life‐history strategies were further evident in resource allocation: under the stress of mixed cues, A1 strongly increased investment in yolk reserves (+76.3%) while suppressing somatic growth (−11.5%), whereas A5 showed a slight, non‐significant reduction in yolk investment (−10.6%) while maintaining positive growth (+14.7%).

### 3.2 Principal Component Analysis of Phenotypic Responses

**FIGURE 2:**
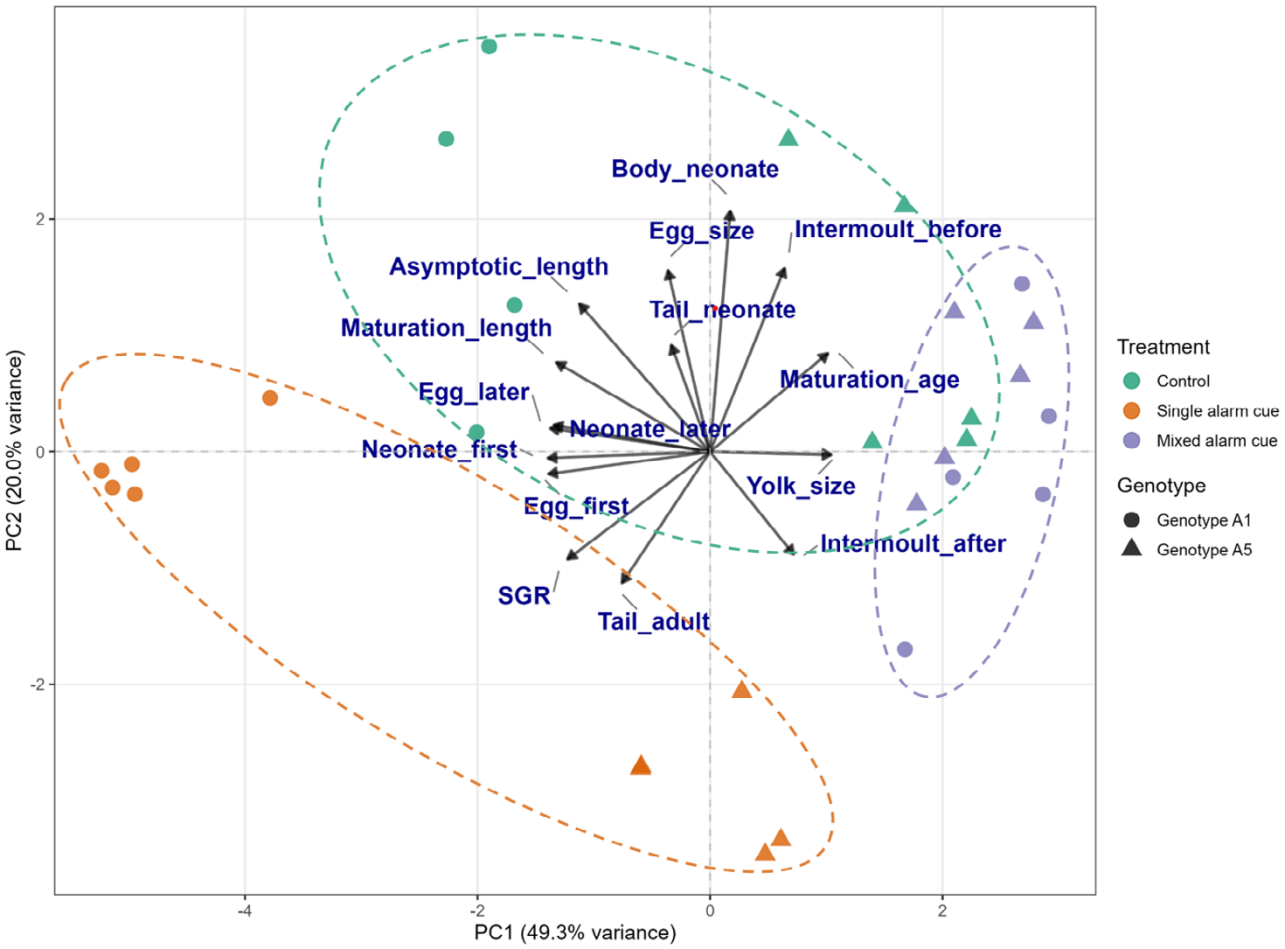
Principal component analysis (PCA) on 15 life‐history traits measured across three treatment conditions (Control, Single alarm cue, Mixed alarm cue) for two *Daphnia* cf. *pulex* genotypes (A1, A5). The treatment‐genotype combinations were shown by the 95% confidence ellipses. Maturation length: Maturation body length; Intermoult before: Intermoult duration before maturation; Intermoult after: Intermoult duration after maturation; Egg first: Egg number of the first clutch; Egg later: Mean egg number of the second and third clutch; Neonate first: Neonate number of the first clutch; Neonate later: Mean neonate number of the second and third clutch; Egg size: Mean egg volume of the first three clutches; Yolk size: Yolk‐to‐egg volume ratio in the first three clutches; Body neonate: Mean neonate size of the first three clutches; Tail neonate: Relative tail spine length of the newly born neonate; Tail adult: Relative tail spine length of the first adult instar; Asymptotic length: Asymptotic body length.

Principal component analysis of 15 life‐history traits revealed distinct multivariate differentiation among treatment groups. The first two principal components explained 69.2% of total variance (Figure 2). PC1 was associated with growth and reproductive traits, representing a continuum from fast‐growth, high‐reproduction to slow‐growth, low‐reproduction strategies (Figure S3). PC2 was associated with developmental timing and morphological traits.

Control samples clustered centrally, while samples exposed to alarm cues showed directional shifts away from this cluster, as evidenced by the minimal overlap of their 95% confidence ellipses. Under the single alarm cue, genotype A1 was primarily separated from controls along PC1, while genotype A5 was separated along PC2. In contrast, under the mixed alarm cue, samples from both genotypes showed a pronounced and common shift along PC1, with A1 displaying a particularly wide spread along this axis. Within this shared displacement, the two genotypes (A1 and A5) formed overlapping clusters, indicating that phenotypic differentiation between genotypes was reduced under mixed cues compared to control or single cue conditions.

### 3.3 Genotype‐Specific Competition Dynamics

**FIGURE 3:**
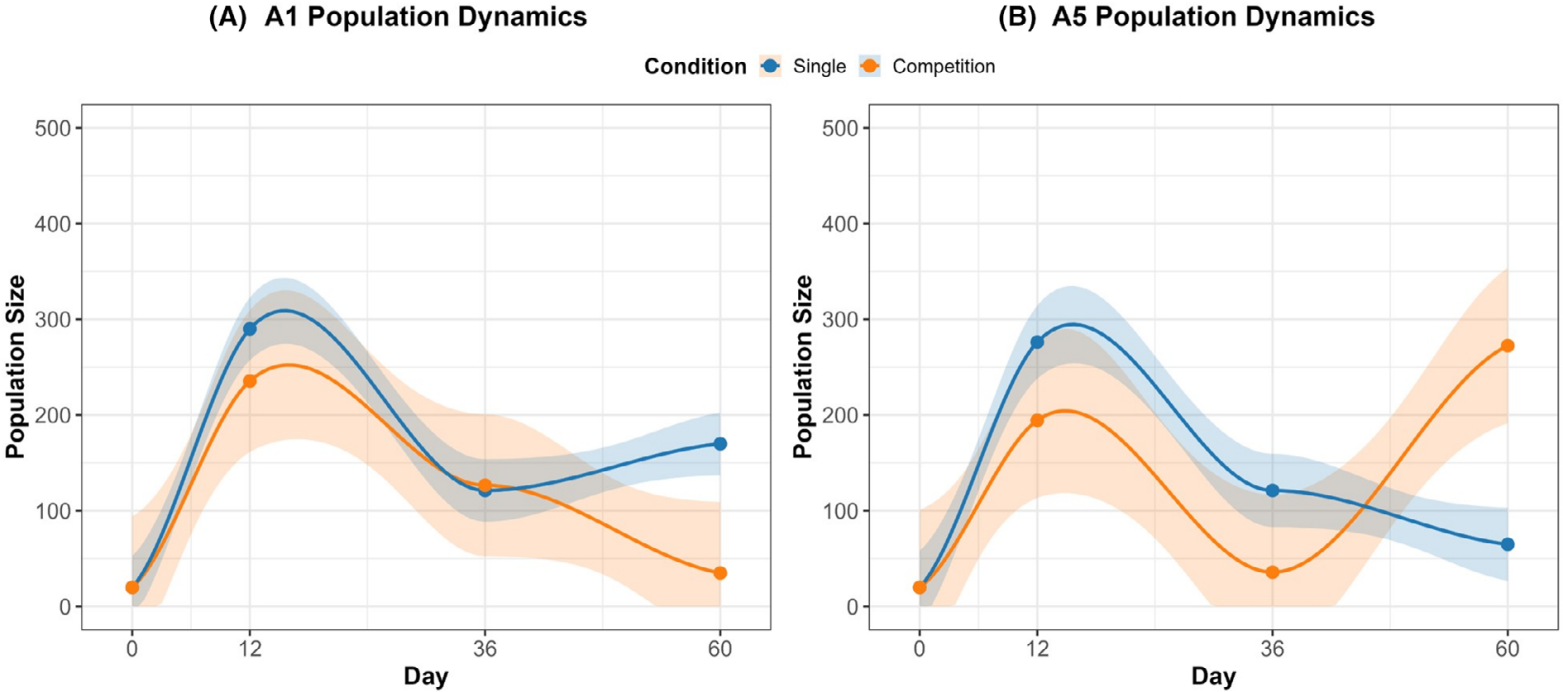
Population trajectories of *Daphnia* genotypes A1 (A) and A5 (B) under single and competitive conditions over a 60‐day period. In the panel, average numbers and 95% confidence interval are shown by line and shade.

Population growth trajectories exhibited distinct nonlinear patterns characterized by an initial rapid increase, followed by a decline, and subsequent recovery (Figure 3). Genotype‐specific responses to competition were evident throughout the experimental period. In single culture, both genotypes showed similar overall growth trajectories, with no consistent difference in population density across the experimental period (Figure 3), consistent with the non‐significant main effect of genotype in the linear mixed‐effects model (Table 3). Under competitive conditions, A5 displayed enhanced growth with population peaks exceeding those in single culture, whereas A1 exhibited marked suppression, particularly during the mid‐to‐late experimental phases (Figure 3B).

**FIGURE 4:**
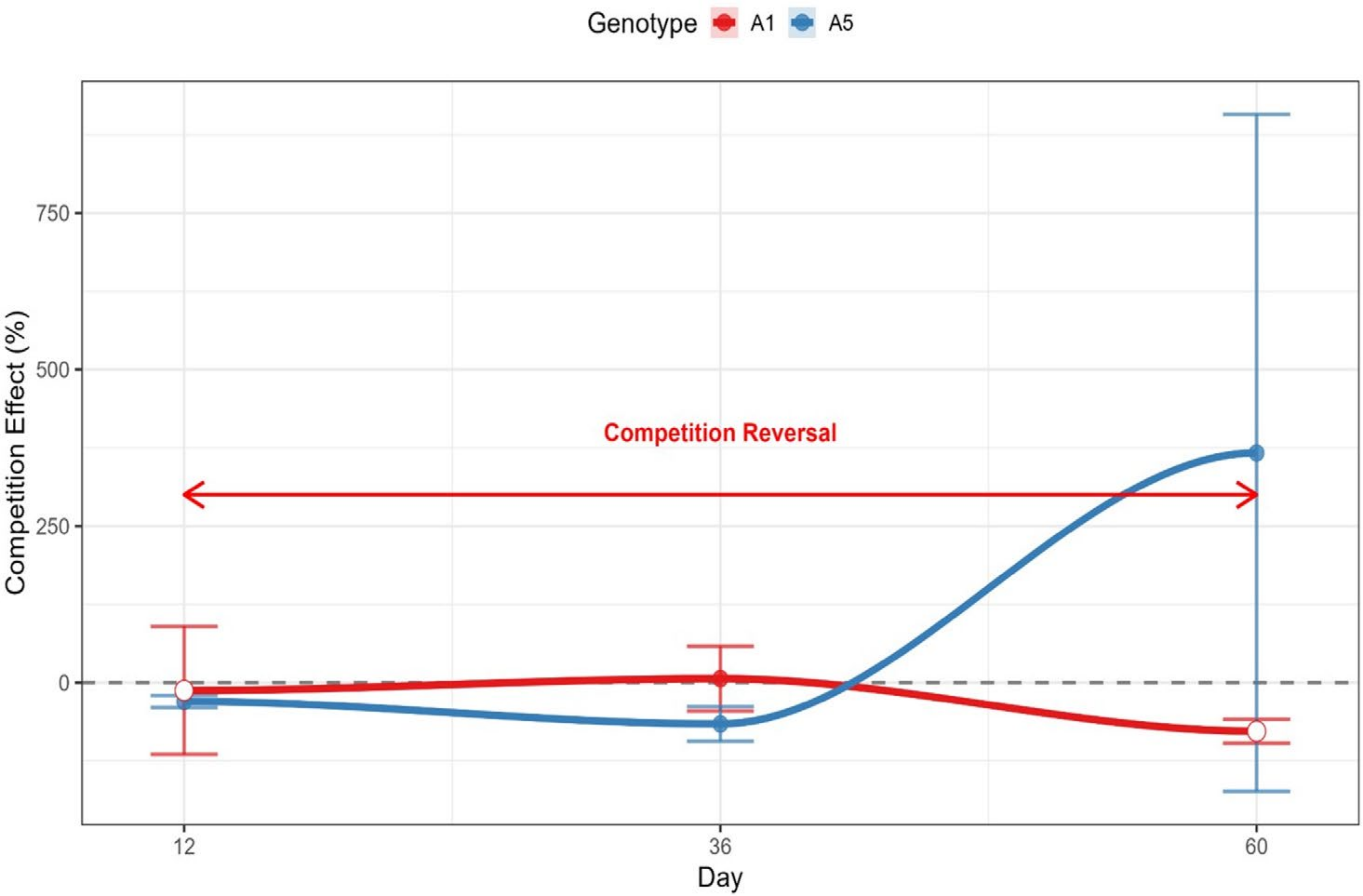
Dynamics in competitive effect sizes between *Daphnia* genotypes.

Analysis of competition effects revealed fundamentally different temporal patterns between A1 and A5 genotypes (Table 2, Figure 4). Genotype A1 initially showed a modest, non‐significant negative response to competition at Day 12 (−14.3%, *t* = 0.712, *p* = 0.503), which remained non‐significant at Day 36 (+6.6%, *t* = −0.355, *p* = 0.735), before declining significantly to −76.2% at Day 60 (*t* = 5.719, *p* = 0.007) (Table 2). In contrast, A5 exhibited a consistent negative competitive response, with moderate effects at Day 12 (−28.1%, *t* = 2.201, *p* = 0.070) that became significant at Day 36 (−68.4%, *t* = 3.324, *p* = 0.016). Notably, by Day 60, A5 demonstrated a large positive effect (+323.1%, *t* = −2.657, *p* = 0.038), indicating a significant competitive advantage at the final time point. These results illustrate divergent and time‐dependent competitive outcomes between the two genotypes, with A5 showing a stronger competitive suppression in mid‐phase and a competitive reversal in the late phase, while A1 showed severe competitive suppression only at the final time point.

**TABLE 2:** Comparison of competition effects at each sampling time for two *Daphnia* genotypes: Net Competition Effect (%) = [(Population size in competition—Mean population size in single culture) – Mean population size in single culture] × 100. A positive value indicates a population size in competition that is larger than in single culture. *p*‐values are from independent samples *t*‐tests comparing population sizes between single culture and competition within each genotype (A1 and A5, analyzed separately) at the same time point. Significant differences are shown in bold.

Genotype	Day	Population size in single culture	Population size in competition culture	Effect size	*t*	*p*
A1	12	290.0 ± 20.5	235.5 ± 73	−14.30	0.712	0.503
A1	36	121.2 ± 5.3	126.6 ± 13.9	6.60	−0.355	0.735
A1	60	170.0 ± 22.4	35.0 ± 5.5	−76.20	5.719	**0.007**
A5	12	276.2 ± 25.7	194.5 ± 25.9	−28.10	2.201	0.070
A5	36	121.1 ± 24.6	35.9 ± 7.5	−68.40	3.324	**0.016**
A5	60	65.0 ± 11.2	272.5 ± 71.3	323.10	−2.657	**0.038**

The linear mixed‐effects model provided a good fit to the population data, explaining 43.9% of the variance in population dynamics (marginal *R* ^2^ = 0.439). Analysis revealed a significant three‐way interaction between genotype, condition, and time (Genotype × Condition × Day: *t* = 3.396, *p* = 0.002; Table 3), indicating that the temporal dynamics of genotype‐specific responses to competition were fundamentally divergent. A significant two‐way Genotype × Condition interaction was also detected (*t* = −2.221, *p* = 0.032), while the Condition × Day interaction approached significance (*t* = −1.988, *p* = 0.050), further supporting the dependence of competitive outcomes on both genotype identity and time. In contrast, the main effects of genotype, condition, and day alone were not statistically significant (*p* > 0.05), underscoring that the key biological signal is encapsulated within these interaction terms, particularly the three‐way effect.

**TABLE 3:** Linear mixed‐effects model with the effects of genotype (A1 or A5), condition (single or competition), Day (12, 36, 60), and their interaction on population size. Significant *p*‐values are shown in bold.

Term	Estimate	SE	df	*t*	*p*	95% CI
Intercept	2.435	0.161	40	15.161	**< 0.001**	2.120, 2.749
Genotype	0.144	0.227	40	0.632	0.531	−0.302, 0.589
Condition	0.123	0.227	40	0.542	0.591	−0.322, 0.568
Day	−0.005	0.004	40	−1.268	0.212	−0.013, 0.003
Genotype: condition	−0.713	0.321	40	−2.221	**0.032**	−1.343, −0.084
Genotype: day	−0.008	0.006	40	−1.512	0.138	−0.019, 0.002
Condition: day	−0.011	0.006	40	−1.988	**0.050**	−0.022, −0.000
Genotype: condition: day	0.027	0.008	40	3.396	**0.002**	0.011, 0.042

### 3.4 Associations Between Life‐History Traits and Competitive Outcomes

We analyzed nine traits showing significant genotype × treatment (mixed alarm cues) interactions: maturation age, maturation length, intermoult interval after maturation, early and late egg production, early and later neonate production, yolk size, and asymptotic length (Table S1). To quantify the association between these trait differences and competitive outcomes, we calculated a composite relevance score (|Trait difference| × |Competition difference| /100) and assessed directional consistency between trait differences (A1—A5) and the competition effect difference (A1—A5 = −444%).

**FIGURE 5:**
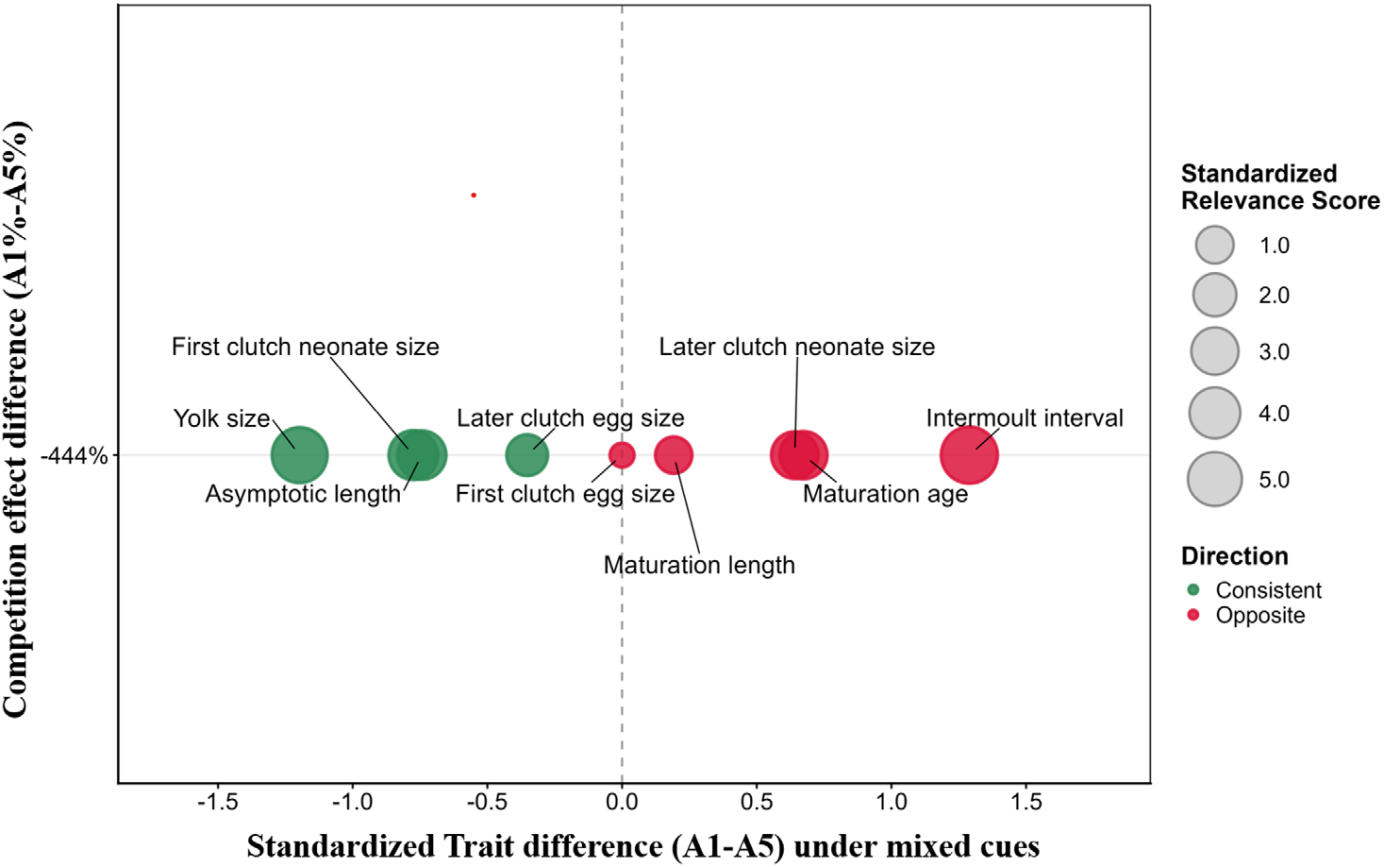
Relation between trait differences between genotypes under mixed cues and competition effect differences between genotypes at day 60. Red circle represents consistent direction, while green one represents opposite direction. Circle size represents the relevance score. (Maturation length: Maturation body length; Intermoult after: Intermoult duration after maturation; Egg first: Egg number of the first clutch; Egg later: Mean egg number of the second and third clutch; Neonate first: Neonate number of the first clutch; Neonate later: Mean neonate number of the second and third clutch; Yolk size: Yolk‐to‐egg volume ratio in the first three clutches; Asymptotic length: Asymptotic body length).

Four of the nine traits showed direction‐consistent relationships, where traits with higher values in A5 were associated with its competitive advantage: intermoult interval after maturation (score = 5.72), yolk size (score = 5.31), early neonate production (score = 3.44), and asymptotic body length (score = 3.30) (Figure 5). The remaining five traits showed direction‐opposite relationships, where traits with higher values in A1 were paradoxically associated with A5's competitive superiority: maturation age (score = 2.99), later neonate production (score = 2.85), later egg production (score = 1.56), maturation body length (score = 0.85), and early egg production (score = 0.00) (Figure 5). In addition, among the nine traits, none showed a statistically significant difference between A1 and A5 under mixed alarm cues after FDR correction for multiple comparisons (all adjusted *p* > 0.05; Table S2).

## 4 Discussion

Our study examines how alarm cue complexity influences life‐history strategies, revealing genotype‐dependent responses that range from adaptive plasticity to more constrained phenotypes. The most notable finding is a shift in life‐history expression: while a single, genotype‐specific cue triggered responses consistent with adaptive plasticity theory, exposure to mixed cues led to more pronounced, genotype‐specific phenotypic changes. These changes were associated with differential competitive outcomes in a related population experiment. This pattern suggests that genetic background plays a critical role in how organisms process uncertain risk information, potentially leading to divergent fitness strategies.

Under the single alarm cue treatment, both genotypes displayed the canonical response predicted by life‐history theory under increased fish predation risk (Boersma et al. 1998; Lass and Spaak 2003; Stoks et al. 2016). The increase in specific growth rate (SGR) likely represents a strategic acceleration to reach a size refuge more quickly, a process potentially mediated by hormonal shifts that shorten intermolt duration and prioritize somatic growth. The associated increase in early reproductive investment in A5 exemplifies a classic bet‐hedging strategy, securing fitness returns in the face of potential early mortality (Lynch 1980; Watts and Tenhumberg 2021; Tian et al. 2022). The genotype‐specific nuances, A1's investment in a morphological defense (longer tail spine) versus A5's investment in fecundity and ultimate size, highlight the existence of multiple, genetically encoded tactical solutions within the overarching strategic shift (Dennis et al. 2011; Stoks et al. 2016). This variation underscores the raw material for evolution within populations facing predictable threats.

The response to mixed alarm cues, however, is inconsistent with a simple escalation of the same adaptive strategy. Instead, it suggests a qualitatively different state driven by high uncertainty. A cue environment composed of alarm signals from multiple genetic backgrounds may not trigger a coordinated life‐history adjustment but rather a chronic, stress‐mediated response. This aligns with the understanding that exposure to multiple, potentially conflicting threat signals can induce a state of high uncertainty and chronic stress, rather than a clear adaptive shift (Relyea 2003; Resetarits Jr et al. 2021). This state, potentially maintained by elevated stress hormone titers, is energetically expensive, diverting resources from growth and reproduction toward maintenance (Stearns 1992), heightened vigilance, and the production of multiple, possibly conflicting, defensive phenotypes (e.g., increased neonate size but reduced asymptotic length) (Isah 2019). The severe, widespread suppression in A1, including a drastic 54.8% delay in maturation and > 50% reductions in fecundity, reflects a conservative “risk‐spreading” strategy, effectively hitting the pause button on development and reproduction in a seemingly unpredictable and high‐threat environment. Conversely, A5's lower responsiveness, maintaining a high SGR and showing only moderate reproductive suppression, may reflect a higher tolerance threshold or a fundamentally different physiological calibration to uncertainty, although the underlying mechanisms remain to be determined. The PCA clearly captures this shift: the single cue induced displacement along a developmental/morphological axis (PC2), while the mixed cue, especially for A5, drove the most pronounced shift along the primary growth‐reproduction continuum (PC1), pulling phenotypes toward a slow‐growth, low‐reproduction pole.

A critical consideration raised by our findings is their relevance to natural systems, where sexually reproducing individuals are genetically distinct and thus likely perpetually exposed to ‘mixed’ cues. Our results are not contradictory to field observations of canonical anti‐predator plasticity but instead refine our understanding of the conditions under which such plasticity is expressed (Dall et al. 2005). We propose that the “single‐cue” response represents an optimal, fine‐tuned strategy when reliable information about a prevailing threat is available (e.g., within a localized patch). The “mixed‐cue” response, conversely, reflects a strategy for coping with informational uncertainty, which may be the default state over larger spatial scales or when integrating signals over time (English et al. 2016). The fact that we observe canonical responses in the wild suggests that organisms frequently encounter situations where the signal is sufficiently clear to trigger them. Our study demonstrates that the capacity for this fine‐tuned response is genetically variable and can be suppressed by informational noise. This creates a scenario of condition‐dependent fitness: genotype A1's strategy excels under clear threat indicators (single cue), while A5's lower responsiveness confers an advantage under ambiguous conditions (mixed cue). Spatial and temporal heterogeneity in predation pressure and cue composition would thus maintain both genotypes (and their respective strategies) in the population, contributing to the maintenance of intraspecific biodiversity through balancing selection (Bolnick et al. 2011). Far from negating the adaptive value of alarm cues, our work highlights how the very structure of information in the environment acts as a selective filter shaping life‐history variation and competitive outcomes.

In addition, our study explores potential connections between the observed life‐history responses and competitive dynamics by integrating our cue exposure assay with a reanalysis of population‐level competition data (Tian et al. 2022). While the two experiments employed different designs and resource levels, they collectively provide a functional link by demonstrating how divergent life‐history responses to mixed alarm cues can translate into shifts in population‐level outcomes. The competition experiment, in which the chemical environment was naturally shaped by mortality within competing populations, offers a complementary perspective: it suggests that phenotypes akin to those induced by mixed alarm cues in our controlled assay, particularly those favoring sustained somatic growth and physiological turnover, may confer a competitive advantage under resource‐limited, density‐dependent conditions (Mueller et al. 1991). Specifically, the less responsive phenotype displayed by genotype A5 under mixed cues, characterized by maintained developmental timing and a faster post‐maturation growth tempo, is consistent with its competitive performance, where it eventually matched or surpassed the initially dominant A1. Conversely, the broadly suppressive response of A1 under mixed cues correlates with its diminished competitive performance in the later stages of population growth.

To explore potential links between life‐history divergence and competitive outcomes, we employed a composite Relevance Score across the nine traits showing significant Genotype × Mixed‐cue interactions. We emphasize at the outset that after FDR correction, none of these traits differed significantly between A1 and A5 under mixed cues (all adjusted *p* > 0.05, Table S2), and PCA revealed largely overlapping genotype clusters under this treatment (Figure 2). The Relevance Score is therefore treated strictly as a hypothesis‐generating ranking of effect sizes, not as evidence for trait‐level mechanisms. Despite this null result at the level of individual traits, the ranking yields a striking pattern. The highest‐scoring traits were intermoult interval after maturation (5.72), yolk size (5.31), first‐clutch neonate size (3.44), asymptotic length (3.30), and maturation age (2.99). What unites the top five is not a single axis of life‐history variation but a shared connection to resource acquisition and processing tempo rather than to terminal allocation endpoints. Intermoult interval governs the frequency of resource assimilation episodes in indeterminately growing crustaceans (Atwood and Sandeman 1982). Yolk size sets the initial capital endowment of offspring (Glazier 1992), and first‐clutch neonate size and asymptotic length represent early and late outcomes of the same somatic growth trajectory. In contrast, the lower‐ranking traits (maturation age, late reproduction) are closer to classical “decisions” about developmental timing and reproductive termination, traits that, under the convergent suppression imposed by mixed cues, appear to vary too little between genotypes to drive competitive differences.

This pattern carries an important implication. The absence of significant static trait differences under mixed cues, combined with overlapping PCA ellipses, suggests that the competitive reversal at Day 60 is unlikely to be explained by divergent mean phenotypes. Instead, we propose that the reversal may arise from differences in dynamic resource routing, specifically, how quickly each genotype rebuilds soma after each molting event, and how it partitions assimilate between late reproduction and somatic maintenance under sustained cue uncertainty. Such processes operate at the scale of individual instar cycles and are invisible to endpoint ANOVAs on static trait means. This interpretation is consistent with a growing body of work showing that competitive outcomes in size‐structured populations often depend more on growth trajectories and instar‐specific allocation rules than on final mean trait values (Persson et al. 1998; De Roos et al. 2003). We therefore refrain from assigning a pivotal mechanistic role to any single trait. Nevertheless, the ranking is consistent with a working hypothesis: genotypes that better sustain somatic tempo and early offspring provisioning (shorter intermoult, larger initial yolk/neonate size) may, under density‐dependent competition, translate a converged “slow‐growth” baseline into a small but compounding turnover advantage over successive instars. This pattern provides a clear, mechanistically motivated target for future work, including time‐series instar tracking and molt‐resolved growth measurements.

These findings integrate and extend several theoretical frameworks. They move beyond models of predator‐induced plasticity by highlighting risk uncertainty as a distinct selective agent that can override or invert adaptive responses to single risks (Ferrari et al. 2010). Our results align with the concept of “multidimensional stress landscapes,” where conflicting cues can lead to maladaptive or constrained phenotypes (Relyea 2003; Resetarits Jr et al. 2021). The strong genotype‐by‐environment effects underscore the importance of intraspecific variation in how organisms integrate and respond to complex stress. Here, the divergent strategies of genotypes A1 (broad suppression) and A5 (strategic reallocation) can be understood as alternative “coping styles” (reactive vs. proactive) within an organismal integrated stress response framework. Their fitness consequences are entirely dependent on the environmental context (single vs. mixed threat), demonstrating that the success of a given stress response syndrome is context‐dependent. This genetic variation in stress response strategies is essential for population resilience in fluctuating environments (Mojica and Kültz 2022).

In conclusion, our study demonstrates that environmental uncertainty, encoded by conflicting intraspecific alarm cues, does not merely heighten risk perception but fundamentally alters life‐history strategies. It shifts responses from an efficient, threat‐specific “life‐history switch” to a state of chronic stress managed in genotype‐specific ways, either as a conservative shutdown (A1) or a strategic reallocation of life‐history effort (A5). Crucially, the success of a given strategy is entirely contingent on the match between a genotype's stress‐coping syndrome and the informational context. This context‐dependent fitness leads to a substantial shift in competitive outcomes, as evidenced by the reversal of dominance hierarchies under conditions mimicking complex information. Thus, signals from dying competitors can act as a form of **“**chemical interference” that reshapes phenotypes and, consequently, the dynamics of intraspecific competition. This reveals a novel pathway by which the very nature of information, its clarity or ambiguity, serves as a selective sieve, helping to maintain a portfolio of response strategies that underpins population resilience in unpredictable environments.

Our findings, while clear under controlled conditions, come with inherent limitations that define the scope of our current inferences and chart a course for future research. First, we note that the mixed alarm treatment contained half the concentration of each genotype's own cue relative to the single treatment, which may have influenced the results. Future studies with appropriate concentration controls would help clarify this point. Second is the focus on a limited set of alarm cues in a controlled laboratory setting. Furthermore, the alarm cue experiment and the competition assay were conducted under different food concentrations, which limits the direct comparability between the two datasets. Addressing this limitation requires moving toward more ecologically realistic conditions. In natural systems, predation risk assessment involves integrating multiple, and sometimes conflicting, cues within a complex information matrix that includes kairomones, resource levels, and conspecific density (Ferrari et al. 2010). Future work should test these responses in semi‐natural mesocosms with live predators to determine the in situ fitness consequences of these plastic strategies, bridging the gap between laboratory mechanisms and field relevance (e.g., Relyea 2003; Resetarits Jr et al. 2021). Mechanistically, direct measurement of metabolic rate and key hormonal pathways (e.g., ecdysteroids, juvenile hormone) is needed to validate the proposed model of energy reallocation (Zera and Harshman 2001). Moreover, a comparative transcriptomic analysis of genotypes A1 and A5 would be powerful for identifying the genetic regulatory networks that decode cue complexity and orchestrate these divergent strategies, potentially illuminating the role of conserved stress response pathways like the integrated stress response (Pakos‐Zebrucka et al. 2016). Ultimately, our study demonstrates that the nature of a threat, its clarity or ambiguity, is as important as its presence, fundamentally reshaping life‐history traits, altering competitive balances, and maintaining the genetic variation upon which natural selection acts.

## Author Contributions

**Xiaofei Tian:** conceptualization (equal), formal analysis (equal), investigation (equal), methodology (equal), supervision (equal), writing – original draft (equal), writing – review and editing (equal). **Qi Zhang:** investigation (equal). **Cheng Li:** investigation (equal). **Wenping Feng:** investigation (equal), methodology (equal), writing – original draft (equal), writing – review and editing (equal). **Xiumei Zhang:** supervision (equal).

## Conflicts of Interest

The authors declare no conflicts of interest.

## Supporting information


**Data S1:** Abundance (individual number/bottle) of D. cf. pulex JPN1 genotypes in single and multiple genotypes runs for 60 days.

**Table S1:** The results of two‐way ANOVA with the effects of genotype, alarm cue treatment (single and control, and mix and control, respectively), and their interaction on phenotypic traits in *Daphnia pulex* . Statistically significant genotype × mix alarm treatment interactions are shown in bold red. Significant *p*‐values are shown in bold.
**Table S2:** Phenotypic differences for traits with a significant Genotype × Treatment interaction between *Daphnia pulex* genotypes A1 and A5 under mixed alarm cue exposure. Significant *p*‐values are shown in bold.
**Figure S1:** Genotype‐specific plastic responses of *Daphnia* life history traits to different alarm cue environments. The panel displays trait responses (e.g., growth, reproduction, morphology) of two clonal *Daphnia* genotypes (A1 and A5) under control, conspecific alarm cue, and mixed alarm cue conditions. Color denotes treatment (gray: Control; blue: Alarm single; orange: Alarm mix). Filled/open boxes represent genotypes A1/A5, respectively. Within each genotype, different letters (a, b) indicate significant treatment differences; within each treatment, different letters (x, y) indicate significant genotype differences. Same letters indicate no significant difference (FDR‐corrected, *p* > 0.05). (First clutch neonate no.: Neonate number of the first clutch; Later clutch neonate no.: Mean neonate number of the second and third clutch; Neonate size: Mean neonate size of the first three clutches; Neonate tail spine ratio: Relative tail spine length of the newly born neonate; Adult tail spine ratio: Relative tail spine length of the first adult instar; Asymptotic length: Asymptotic body length).
**Figure S2:** Proportional changes in life history traits of *Daphnia* genotypes in response to mixed‐species alarm cues (left panel) and conspecific alarm cues (right panel). Values represent the percentage change in mean trait value relative to the control treatment [(Treatment mean − Control mean)/Control mean × 100%]. Red tiles indicate trait enhancement (positive change), blue tiles indicate trait suppression (negative change), and white tiles indicate minimal change. Numerical labels show the exact percentage change. *n* = 5 per genotype × treatment combination.
**Figure S3:** Variable loadings and trait contributions to principal components (PC1 and PC2). Heatmap showing (A) the association of each life‐history trait with PC1 and PC2 axes (variable loadings), and (B) the percentage contribution of each trait to the explained variance of the first two principal components.

## Data Availability

All trait data analyzed in this study have been deposited on Figshare and are publicly available at: https://figshare.com/s/b756496aba7949b62bd5. Data of the competition experiments are provided as an additional file.
