# Supplementary material for: What the Dying are Saying to the Living: Mixed Conspecific Alarm Cues Reverse Competitive Hierarchies by Reshaping Genotype‐Specific Life Histories
Source: Ecol Evol. 2026 Sep 13;16(9):e74356. doi: 10.1002/ece3.74356 (PMC13572202; doi:10.1002/ece3.74356)
Supplement: Supplementary file 1 [file ECE3-16-e74356-s001.docx]

Supporting information

**Title: What the dying are saying to the living: mixed conspecific alarm cues reverse competitive hierarchies by reshaping genotype-specific life histories**

**Supplementary Methods**

1.**Detailed competition experimental protocol from Tian et al. (2022)**

The competition experiment from which data were analyzed was conducted as follows. Experimental units were 1 L bottles containing COMBO medium and the green alga *Scenedesmus obliquus* as a food source. Three types of population runs were established: (i) single-genotype controls (20 individuals of either A1 or A5), (ii) two-genotype competition (10 individuals each of A1 and A5), and (iii) four-genotype competition (not used in the present study). Each treatment had four replicate bottles. Populations were maintained in a climate-controlled room at 20°C with a 14:10 light:dark cycle. Bottles were gently swirled daily to prevent settling. Every third day, 20% of the medium was replaced, and a concentrated algal suspension was added to replenish food, maintaining an approximate average food concentration of 0.33 mg C L⁻¹ day⁻¹. On census days (0, 12, 36, 60), all individuals from a bottle were collected, counted, and a subsample was preserved in 95% ethanol for subsequent genetic identification. Genotype identification was performed via polymerase chain reaction (PCR) and sequencing of mitochondrial 12S rRNA and control region sequences, as detailed in Tian et al. (2022).

**References**

Tian X., Ohtsuki H., Urabe J. 2022. Competitive consequences determined by phenotypic but not genetic distance: A study with asexual water flea genotypes. Funct. Ecol. 36: 2152-2162.

Table S1 The results of two-way ANOVA with the effects of genotype, alarm cue treatment (single and control, and mix and control, respectively), and their interaction on phenotypic traits in *Daphnia pulex*. Statistically significant genotype × mix alarm treatment interactions are shown in bold red. All p-values are FDR-corrected (Benjamini-Hochberg procedure). Significant P-values are shown in bold.

|  |  | Genotype (G) | | Cue treatment (Ct) | | G × Ct | |
| --- | --- | --- | --- | --- | --- | --- | --- |
| Trait | Treatment | F-value | *p* | F-value | *p* | F-value | *p* |
| Maturation age (days) | Single and control | 10.667 | **0.005** | 29.630 | **<0.001** | 4.741 | **0.045** |
|  | Mix and control | 0.173 | 0.683 | 5.558 | **0.031** | 5.558 | **0.031** |
| Maturation body length (mm) | Single and control | 112.288 | **<0.001** | 0.244 | 0.628 | 2.933 | 0.106 |
|  | Mix and control | 15.565 | **0.001** | 34.926 | **<0.001** | 12.758 | **0.003** |
| Intermoult duration before maturation (days) | Single and control | 1.919 | 0.185 | 26.655 | **<0.001** | 2.497 | 0.134 |
|  | Mix and control | 0.695 | 0.417 | 0.229 | 0.639 | 2.855 | 0.110 |
| Intermoult duration after maturation (days) | Single and control | 7.034 | **0.017** | 0.957 | 0.342 | 5.846 | **0.028** |
|  | Mix and control | 5.157 | **0.037** | 8.832 | **0.009** | 6.006 | **0.026** |
| SGR (day^-1^) | Single and control | 16.988 | **0.001** | 58.283 | **<0.001** | 1.233 | 0.283 |
|  | Mix and control | 8.477 | **0.010** | 0.007 | 0.934 | 3.202 | 0.092 |
| Egg number of the first clutch | Single and control | 36 | **<0.001** | 21.777 | **<0.001** | 1.777 | 0.201 |
|  | Mix and control | 7.000 | **0.018** | 41.286 | **<0.001** | 7.000 | **0.018** |
| Mean egg number of the second and third clutch | Single and control | 102.311 | **<0.001** | 36.213 | **<0.001** | 33.197 | **<0.001** |
|  | Mix and control | 4.891 | **0.042** | 23.674 | **<0.001** | 7.848 | **0.013** |
| Neonate number of the first clutch | Single and control | 55.125 | **<0.001** | 28.125 | **<0.001** | 3.125 | 0.096 |
|  | Mix and control | 5.538 | **0.032** | 30.154 | **<0.001** | 15.385 | **0.001** |
| Mean neonate number of the second and third clutch | Single and control | 137.619 | **<0.001** | 42.076 | **<0.001** | 38.571 | **<0.001** |
|  | Mix and control | 12.522 | **0.003** | 14.696 | **0.001** | 5.565 | **0.031** |
| Mean egg volume of the first three clutches (mm^3^) | Single and control | 3.983 | 0.063 | 1.473 | 0.242 | 0.377 | 0.548 |
|  | Mix and control | 0.303 | 0.590 | 0.697 | 0.416 | 0.365 | 0.554 |
| Yolk-to-egg volume ratio in the first three clutches | Single and control | 56.589 | **<0.001** | 5.610 | **0.031** | 18.769 | **0.001** |
|  | Mix and control | 56.172 | **<0.001** | 3.403 | 0.084 | 14.670 | **0.001** |
| Mean neonate size of the first three clutches (mm) | Single and control | 15.937 | **0.001** | 41.342 | **<0.001** | 6.212 | **0.024** |
|  | Mix and control | 0.078 | 0.783 | 1.560 | 0.230 | 2.384 | 0.142 |
| Relative tail spine length of the newly born neonate | Single and control | 6.883 | **0.018** | 5.512 | **0.032** | 0.003 | 0.954 |
|  | Mix and control | 3.146 | 0.095 | 3.413 | 0.083 | 0.607 | 0.447 |
| Relative tail spine length of the first adult instar | Single and control | 6.958 | **0.019** | 15.500 | **0.001** | 2.164 | 0.162 |
|  | Mix and control | 1.625 | 0.222 | 0.381 | 0.546 | 2.465 | 0.137 |
| Asymptotic body length (mm) | Single and control | 86.313 | **<0.001** | 10.290 | **0.005** | 16.950 | **0.001** |
|  | Mix and control | 3.042 | 0.100 | 56.014 | **<0.001** | 8.724 | **0.009** |

Table S2 Phenotypic differences for traits with a significant Genotype × Treatment interaction between *Daphnia pulex* genotypes A1 and A5 under mixed alarm cue exposure. All p-values are FDR-corrected (Benjamini-Hochberg procedure). Significant P-values are shown in bold.

| Trait | A1 | A5 | Trait difference | t value | df | p |
| --- | --- | --- | --- | --- | --- | --- |
| Maturation age (days) | 9.6 ± 1.28 | 8.2 ± 0.2 | 1.4 | 1.074 | 8 | 0.471 |
| Maturation body length (mm) | 1.29 ± 0.03 | 1.28 ± 0.02 | 0.009 | 0.287 | 8 | 0.879 |
| Intermoult duration after maturation (days) | 2.787 ± 0.17 | 2.33 ± 0 | 0.453 | 2.619 | 8 | 0.186 |
| Egg number of the first clutch | 1.6 ± 0.24 | 1.6 ± 0.24 | 0 | 0 | 8 | 1.000 |
| Mean egg number of the second and third clutch | 2.0 ± 0.35 | 2.2 ± 0.12 | -0.2 | -0.535 | 8 | 0.782 |
| Neonate number of the first clutch | 1.2 ± 0.2 | 1.6 ± 0.24 | -0.4 | -1.265 | 8 | 0.436 |
| Mean neonate number of the second and third clutch | 2.2 ± 0.34 | 1.8 ± 0.2 | 0.4 | 1.016 | 8 | 0.508 |
| Yolk-to-egg volume ratio in the first three clutches | 0.105 ± 0.005 | 0.13 ± 0.01 | -0.029 | -2.3 | 8 | 0.230 |
| Mean neonate size of the first three clutches (mm) | 1.56 ± 0.06 | 1.65 ± 0.04 | -0.091 | -1.205 | 8 | 0.437 |

**Fig. S1** Genotype-specific plastic responses of *Daphnia* life history traits to different alarm cue environments. The panel displays trait responses (e.g., growth, reproduction, morphology) of two clonal *Daphnia* genotypes (A1 and A5) under control, conspecific alarm cue, and mixed alarm cue conditions. Color denotes treatment (gray: Control; blue: Alarm single; orange: Alarm mix). Filled/open boxes represent genotypes A1/A5, respectively. Within each genotype, different letters (a, b) indicate significant treatment differences; within each treatment, different letters (x, y) indicate significant genotype differences. Same letters indicate no significant difference (FDR-corrected, p>0.05). (First clutch neonate no.: Neonate number of the first clutch; Later clutch neonate no.: Mean neonate number of the second and third clutch; Neonate size: Mean neonate size of the first three clutches; Neonate tail spine ratio: Relative tail spine length of the newly born neonate; Adult tail spine ratio: Relative tail spine length of the first adult instar; Asymptotic length: Asymptotic body length).

Fig. S2 Proportional changes in life history traits of *Daphnia* genotypes in response to mixed-species alarm cues (Alarm_mix, left panel) and conspecific alarm cues (Alarm_single, right panel). Values represent the percentage change in mean trait value relative to the control treatment [(Treatment mean − Control mean) / Control mean × 100%]. Red tiles indicate trait enhancement (positive change), blue tiles indicate trait suppression (negative change), and white tiles indicate minimal change. Numerical labels show the exact percentage change. (Maturation length: Maturation body length; Intermoult before: Intermoult duration before maturation; Intermoult after: Intermoult duration after maturation; Egg first: Egg number of the first clutch; Egg later: Mean egg number of the second and third clutch; Neonate first: Neonate number of the first clutch; Neonate later: Mean neonate number of the second and third clutch; Egg size: Mean egg volume of the first three clutches; Yolk size: Yolk-to-egg volume ratio in the first three clutches; Body neonate: Mean neonate size of the first three clutches; Tail neonate: Relative tail spine length of the newly born neonate; Tail adult: Relative tail spine length of the first adult instar; Asymptotic length: Asymptotic body length).

Fig. S3 Variable loadings and trait contributions to principal components (PC1 and PC2). Heatmap showing (A) the association of each life-history trait with PC1 and PC2 axes (variable loadings), and (B) the percentage contribution of each trait to the explained variance of the first two principal components. (Maturation length: Maturation body length; Intermoult before: Intermoult duration before maturation; Intermoult after: Intermoult duration after maturation; Egg first: Egg number of the first clutch; Egg later: Mean egg number of the second and third clutch; Neonate first: Neonate number of the first clutch; Neonate later: Mean neonate number of the second and third clutch; Egg size: Mean egg volume of the first three clutches; Yolk size: Yolk-to-egg volume ratio in the first three clutches; Body neonate: Mean neonate size of the first three clutches; Tail neonate: Relative tail spine length of the newly born neonate; Tail adult: Relative tail spine length of the first adult instar; Asymptotic length: Asymptotic body length).
